# Editorial: Computational systems biomedicine

**DOI:** 10.3389/fgene.2022.1047760

**Published:** 2022-10-12

**Authors:** Richa Batra, Priyanka Baloni, Nicolas Alcaraz, Anne-Christin Hauschild, Alejandra Cervera

**Affiliations:** ^1^ Department of Physiology and Biophysics, Institute for Computational Biomedicine, Englander Institute for Precision Medicine, Weill Cornell Medicine, New York, NY, United States; ^2^ School of Health Sciences, Purdue University, West Lafayette, IN, United States; ^3^ Novo Nordisk Foundation Center for Protein Research, Faculty of Health and Medical Sciences, University of Copenhagen, Copenhagen, Denmark; ^4^ Department of Medical Informatics, University Medical Center Göttingen, Georg-August University of Göttingen, Göttingen, Germany; ^5^ Instituto Nacional de Medicina Genómica (INMEGEN), Mexico City, Mexico

**Keywords:** computatonal biology, cancer, COVID-19, networks, omics

## Introduction

This collection invited research articles with a focus on computational approaches using multi-omic datasets and integrative data analysis to gain systems-level understanding of diseases. The articles published under this collection address different biomedical questions including methods for diagnosis or prognosis of cancer, cancer progression as well as pathogenesis of COVID-19 infection. In the following we discuss the seven articles from this collection grouped by the topics.

### COVID-19

The pandemic caused by the SARS-Cov2 virus has prompted the urgent need for a better molecular understanding of the biological processes involved in the infection and progression of COVID-19. In the following paragraphs we summarize two articles from this collection that described the complexity of COVID-19 infection.

Valikangas et al. identified a COVID-19 specific gene signature using bulk and single-cell RNA-Seq profiles of different respiratory diseases. This signature is enriched in immunoglobulin and hemostasis signals as well as was associated with known drugs and chemical compounds such as the anti-inflammatory theophylline. Moreover, they developed a browsable resource for the community to further explore their results.

Hu et al. developed a pipeline to study the role of genetic predisposition associated with COVID-19 severity by using a multimodal network of genes and polygenic risk scores (PRS) for several demographic variables. In the context of COVID-19 this analysis identified associations between COVID-19-related genes and comorbidities such as hypertension, cerebrovascular disease, and ischemic heart disease. Further, blood levels of *PTPN6* gene was linked to lower genetic risk but associated with many complications during progression of the disease. This framework consists of 7,000 genes and has the potential to expand our knowledge on the genetic predisposition to various complex diseases.

### Cancer

Early diagnosis, personalized treatment, and predicting survival are outstanding challenges in the field of cancer treatment. Over the years several computational approaches have been developed to aid in this process. These approaches need continuous upgrading as technological advancements in molecular profiling and big data initiatives lead to better understanding of disease pathogenesis. In the following paragraphs we summarize three articles from this collection that described novel ways to address these persisting issues in the field.

Using RNA-Seq data from The Cancer Genome Atlas (TCGA), You et al. showed that gene *RAC1* is significantly correlated with patient survival in hepatocellular carcinoma (HCC) and this finding was confirmed using an independent cohort. Moreover, immune cell infiltration was quantified using the immune cell type deconvolution approach and *RAC1* expression was found to be highly correlated with 3 types of macrophages: M0, M1 and M2. Furthermore, an 11-gene immune related signature was generated that could be used to stratify patients into low and high risk groups.

Al-Harazi et al. also worked on HCC to identify a cross-species gene signature of early HCC (eHCC) using human and rat microarray gene expression profiles from NCBI Gene Expression Omnibus (GEO) database. This signature was validated using RNA-Seq data from the TCGA database i.e. across cohort and technology. Their integrated cross-species transcriptomics analysis along with gene networks provided a prognostic risk score for HCC to predict patient outcome.

In their follow up work, Al-Harazi et al. developed a novel network-based algorithm to detect subnetwork markers for patients with colorectal cancer (CRC) using mRNA expression and copy number alterations (CNAs) data from NCBI GEO. A signature of 24 genes with deregulated gene expression as well as altered CNAs was used for building the protein-protein interaction (PPI) network using BisoGenet. Subsequently, this network was split into subnetworks and used to build a model for diagnosing CRC patients. This model was validated on an independent cohort from TCGA and was compared to models built on currently available gene signatures.

### Tools

The scientific community requires novel algorithms and easily implemented tools to interpret and determine the biological meaning of the increasing number of OMICS datasets being produced. In the following paragraph we summarize two articles from this collection that have presented algorithms that can be utilized by the broad biomedical community.

Sudhakar et al. developed an algorithm for **p**ersonalized **i**dentification of dri**v**er **o**ncogenes and **t**umor suppressor genes (PIVOT). To the best of the author’s knowledge theirs is the first, supervised approach to identify oncogenes and tumor suppressor genes using multiple omics including miRNA, copy number variation (CNV), and networks apart from the conventionally used features of gene expression and single nucleotide variants (SNVs). It was reported that evaluating the functional impact of a mutation and using that as a feature in the machine learning model led to significant improvement in the performance. The main challenge in developing such tools is the lack of a gold standard to be able to systematically compare the available methods in the domain. For this reason, the methods are highly sensitive to reference sets used for training of the models.

Mechteridis et al. presented the R implementation of KeyPathwayMiner (KPM), a *de novo* pathway enrichment tool for Cytoscape, with new functions for facilitating data input and support for single-cell datasets. Briefly, KPM algorithm offers condition-specific integration of biological networks with one or more omics datasets such as gene expression profiles and/or methylation profiles. The R ecosystem of this algorithm was used in the context of COVID-19 to show enrichment of deregulated pathways. These pathways included predefined pathways like “Cytokine Signaling in Immune System” as well as *de novo* pathways related to viral infections and immune response.

### Outlook

Overall, this collection showcases several computational solutions to the challenges in biomedical research. However, more work is needed in the direction of utilizing the plethora of omics datasets available to the community to solve outstanding challenges. For instance, applications of omics technologies including epigenomics, metabolomics, proteomics, and metagenomics, algorithms addressing the challenges of heterogeneous presentation of diseases or methods to predict alternate use of existing drugs were missing from this collection. Moreover, a global effort is needed to overcome roadblocks in translating the insights gained using these computational approaches to clinical practices.

